# Acute ischemic gangrene of the rectum: Report of 3 cases and review of literature^☆^

**DOI:** 10.1016/j.ijscr.2013.09.011

**Published:** 2013-09-28

**Authors:** Khawaja Azimuddin, Tal Raphaeli

**Affiliations:** Houston Colon & Rectal Surgery, 1125 Cypress Station Dr., Suite G3, Houston, TX 77090, United States

**Keywords:** Rectal, Ischemia, Gangrene, Proctitis, Spontaneous

## Abstract

**INTRODUCTION:**

Acute ischemia of the rectum resulting in full thickness necrosis is extremely uncommon because of its excellent blood supply.

**PRESENTATION OF CASE:**

We present 3 cases with spontaneous ischemic gangrene of the rectum. All three patients were elderly with atherosclerotic arterial disease and presented with hypotensive shock but in none of these patients we encountered a precipitating factor such as preceding vascular surgery or shock state.

**DISCUSSION:**

A high index of suspicion should be maintained in elderly patients with atherosclerotic disease who present with lower GI symptoms with hypotensive shock and an inflamed rectum on CT scan. Immediate beside proctoscopy should be offered to these patients and if the diagnosis is confirmed these patients should be taken to the operating room immediately. If the entire rectum is found to be gangrenous then an emergency APR should be performed and the perineal wound left open. If the rectum is partially gangrenous then a low anterior resection with Hartman's procedure for diversion is appropriate.

**CONCLUSION:**

Prompt diagnosis and resuscitation followed by immediate surgical intervention is necessary to save these elderly patients.

## 1 Introduction

Ischemic gangrene of the rectum is a rare emergency. Normally, the rectum is very well vascularized and acute spontaneous ischemia of the rectum is an unlikely occurrence.^1–4^ We report a series of 3 cases of acute ischemia of the rectum with frank gangrene and present review of the literature. All three patients were elderly with atherosclerotic arterial disease, presented with hypotensive shock and required emergency life-saving surgical intervention.

## 2 Presentation of cases

### 2.1 DC

**Fig. 1:**
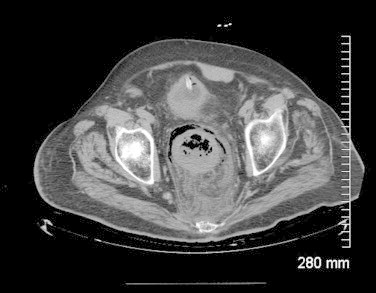
Rectal wall thickening and pneumatosis with perirectal inflammatory stranding.

A 77-year old male with history of hypertension, insulin dependent diabetes, hyperlipidemia, prior stroke and Parkinsonism was admitted from the emergency room with history of bloody diarrhea and lower abdominal pain. He had history of diarrhea off and on for the last 6 weeks and was recently treated for C diff colitis. He was hypotensive and tachycardic and abdominal exam showed marked tenderness in the left lower quadrant. Digital rectal exam revealed dark red blood and chunks of mucosal slough. Investigations revealed WBC of 22,000, acute renal failure and acidosis. CT showed peri rectal stranding and pneumatosis of the rectal wall (Fig. 1). Bedside proctoscopy revealed black and necrotic rectal mucosa, mucosal slough and dark red blood.

Patient was resuscitated with fluids, vasopressors and IV antibiotics were started. He was taken for an emergency exploratory laparotomy. During surgery, complete gangrene of the rectum extending from recto sigmoid junction to the dentate line was found. An abdominoperineal resection of the rectum was performed. The levators were divided close to the rectum since the surgery was done for a non-malignant condition. Once the specimen was exteriorized, a left lower quadrant colostomy was performed and the perineal wound was packed with Kerlex gauze. Five days later, when the patient was hemodynamically stable, he was brought back to the operating room and the perineal wound was closed. Postoperatively, the recovery was uneventful and the patient was discharged to a nursing home on the 17th postoperative day. Histopathological examination of the rectal specimen showed extensive ischemic ulceration and necrosis extending to the muscularis propria.

### 2.2 EK

A 93-year old otherwise very active male was admitted with lethargy and lower abdominal pain. He was constipated recently and took some laxatives, as a result he had diarrhea just before admission. Past medical history was significant for hypertension, atrial fibrillation, stroke and a recent TIA. He also had a sigmoid colectomy in past for cancer. Upon examination he was hypotensive and tachycardic. Abdominal exam showed lower abdominal tenderness and distention. Fecal impaction was found on digital rectal exam.

**Fig. 2:**
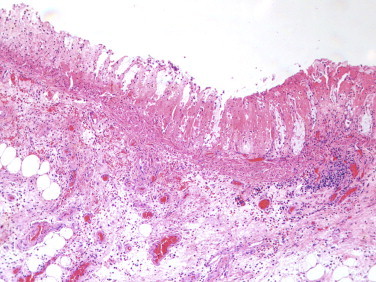
Ischemic necrosis of the mucosa ghost like remnants of glands with loss of the epithelium and bland necrosis of the lamina propria.

White blood count was elevated at 24,000. Lactic acid was elevated. CT scan showed some fluid in the pelvis and mild peri rectal stranding. Patient was taken for an exploratory laparotomy in view of abdominal tenderness, septic shock, leukocytosis and lactic acidosis. After induction of general anesthesia, manual disimpaction of rectum was performed. Very foul smelling pus, stools and blood was encountered. Proctoscopy at this stage revealed a completely black rectum all the way down to the dentate line. During laparotomy a swollen, discolored and inflamed rectum was identified. An abdominoperineal resection of the rectum was performed. The perineal wound was packed with Kerlex gauze. Patient was brought to the operating room on the 5th postoperative day for delayed closure of the perineal wound. After a prolonged and slow recovery he was discharged to a nursing home on the 18th postoperative day. Histopathological exam of the specimen showed severe acute ischemic proctitis (Fig. 2).

### 2.3 GR

**Fig. 3:**
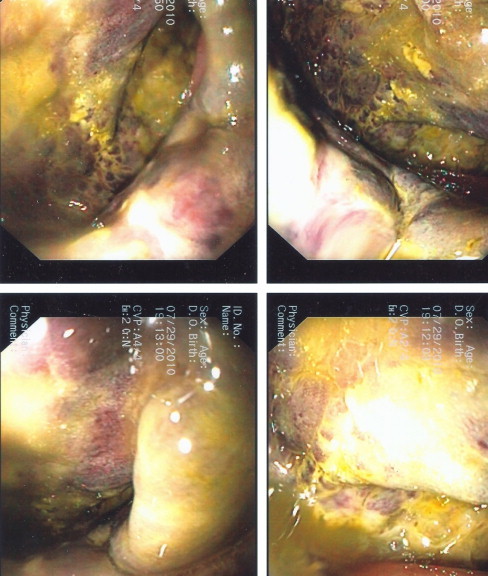
Purplish black and ischemic rectal mucosa. (For interpretation of the references to color in this figure legend, the reader is referred to the web version of this article.)

An 80-year old female was admitted from nursing home with history of bloody diarrhea. Past medical history included hypertension, hyperlipidemia and history of stroke. She was hypotensive and septic. White cell count was 17,000. CT scan showed thickening of the rectal wall. She was started on IV fluids, vasopressors and intravenous antibiotics. A bedside flexible sigmoidoscopy was performed which revealed severe ischemia and necrosis of the rectal mucosa (Fig. 3). Mild ischemic changes extended up to the splenic flexure. After resuscitation an exploratory laparotomy was performed which revealed frank gangrene of almost the entire rectum except the last 2 cm above the dentate line. Low anterior dissection of the rectum was performed all the way down to the levators and anal canal. Here the anorectal stump was stapled off and divided. A near complete proctectomy and Hartman's procedure was performed. Because the anal canal was viable, we were able to avoid an abdominoperineal resection of the rectum. The abdomen was washed with copious amounts of normal saline and large drains were left in the pelvis. Postoperative recovery was slow but uneventful and the patient was finally transferred back to the nursing home. Histopathology showed extensive necrosis of the mucosa extending into the muscularis propria.

## 3 Discussion

Acute rectal ischemia is rare because the rectum has abundant blood supply and rich collaterals. The superior rectal artery which is the terminal branch of inferior mesenteric artery supplies most of the blood to rectum. Some blood is derived from the middle rectal arteries which are branches of the internal iliac artery or the internal pudendal artery and also from the inferior rectal artery which is a terminal branch of the internal iliac artery. There is extensive intramural anastomosis between the branches of these arteries especially in the lower part of the rectum. Extramural collaterals also occur between the lowest sigmoid and superior rectal arteries. This watershed area (Critical Point of Sudeck) is not considered to be critical anymore because adequate collateral are almost always present.^2,3^ In addition collaterals also exist between lumbar vessels and branches of the internal iliac arteries which can maintain circulation through the middle and inferior rectal arteries when iliac vessels are compromised.^4^

In addition to these named branches rectum also receives blood supply from muscular branches of the levator, and some twigs from the inferior vesical or vaginal arteries. This extensive blood supply ensures that rectum is perfused even in cases where the rest of the bowel is compromised.

*Pathophysiology*: Unlike ischemic colitis or proctitis, frank gangrene of the rectum is very rare. It is usually described in elderly patients with significant atherosclerotic disease and cardiac risk factors in the presence of hemodynamic instability. It occurs as a result of sudden acute compromise in blood flow in patients with inadequate collateral circulation around the rectum. Most reported cases in literature involve an acute event such as interruption of inferior mesenteric artery during aortofemoral bypass or ligation of internal iliac vessels in patients with atherosclerotic narrowing of major arteries. Hypotensive shock or a low flow state will compound this problem and the rectum becomes ischemic despite the usually excellent collateral circulation. In none of our patients, a preceding surgical event, hemodynamic compromise or low flow state was encountered.

Other rare causes include angiographic embolization of the blood supply of the rectum, infected strangulated hemorrhoids, vasculitis and or immune complex thrombi related to systemic lupus erythematosis^5,6^ or direct necrotizing effect of phosphate enemas on the rectum.^7^ However, like Reinus et al.,^8^ there was no major precipitating event in any of our three cases.

*Clinical presentation and diagnosis*: Most patients will present with diarrhea and rectal bleeding. Abdominal and pelvic pain and distention is also common. Fever, tachycardia along with hypotension is often present. Lower abdominal tenderness is often present but because of the extra peritoneal pelvic position of the rectum these signs may be delayed. Nelson et al. has reported loss of anal tone as an early sign of acute rectal ischemia.^4^

Leukocytosis and acidosis are reflective of sepsis and shock. CT scan shows rectal wall thickening and peri rectal stranding. Pneumatosis in the rectal wall may also be present in advanced cases as was seen in one of our patients. Later rectal perforation with extra luminal air may be seen. Angiography, though not advisable in acute gangrenous cases, shows diffuse atheromatous disease the aortoilliac vessels with abrupt cut off.

Endoscopic examination of the rectum either using a rigid proctoscope or a bedside flexible scope remains the cornerstone for diagnosis of rectal ischemia. Early findings include erythema, mucosal and sub mucosal hemorrhages, and ulceration. In the setting of lower GI symptoms these changes can be confused with IBD or infectious colitis. With more serious compromise of the blood supply necrosis or frank gangrene of the rectum will be seen. In two of our three cases the rectal wall was black and completely gangrenous.

*Management*: In early cases or in cases with superficial ischemia conservative management is appropriate but in cases of frank gangrene of the rectum, emergency surgery is the only option. While preparations are being made for surgery patient should be resuscitated and started on broad spectrum antibiotics. In the OR patient should be placed in modified lithotomy position with Lloyd Davis stirrups to allow access to the rectum during surgery. Operation should start with a proctoscopy to evaluate the extent of ischemia. Visual inspection of the rectum during surgery will also help determine the extent and level of ischemia. In most cases a complete proctectomy is required. In the emergency situation this is best accomplished by an APR. The perineal wound is packed in order to avoid infection in a closed space and to expedite the operation. The wound can be closed after a few days when patient is stable and the risk of infection has decreased. Proctectomy may also be achieved with intersphincteric dissection and excision of the rectum but is a longer more complex operation. During an emergency operation every effort must be made to abbreviate the operation and get the patient back to ICU for further resuscitation.

In cases where the lower rectum is spared a low anterior resection of the rectum along with a Hartman's procedure may be performed. We do not recommend leaving behind a gangrenous or ischemic segment of the rectum as has been suggested by some.^9^ Rather we support the view of Maun et al. that leaving behind a gangrenous rectal segment will be a source of persistent sepsis and should be removed.^10^ A drain should be left in pelvis and stools be diverted with a colostomy.

## 4 Conclusion

Acute full thickness rectal ischemia with gangrene is a rare clinical entity. Immediate bedside proctoscopy should be considered in elderly patients with atherosclerotic disease who present with lower GI symptoms, hypotensive shock and the CT shows an inflamed and swollen rectum. Conservative management is not appropriate in these cases and preoperative resuscitation followed by emergency surgery to resect the frankly gangrenous bowel is necessary.

## Conflict of interest

None.

## Funding

None.

## Ethical approval

I have obtained written consent from the patient. This can be provided if needed.

## Author contributions

Khawaja Azimuddin: Data and case selection and preparation.

Tal Raphaeli: Writing.
